# First Principles Simulations of Phenol and Methanol Detector Based on Pristine Graphene Nanosheet and Armchair Graphene Nanoribbons

**DOI:** 10.3390/s19122731

**Published:** 2019-06-18

**Authors:** Muhammad Haroon Rashid, Ants Koel, Toomas Rang

**Affiliations:** Thomas Johan Seebeck Department of Electronics, Tallinn University of Technology, Ehitajate tee 5, 12616 Tallinn, Estonia; ants.koel@taltech.ee (A.K.); toomas.rang@taltech.ee (T.R.)

**Keywords:** graphene, graphene nanosheet, armchair graphene nanoribbon, phenol, methanol, detector, photocurrent

## Abstract

Over the last decade graphene based electronic devices have attracted the interest of researchers due to their exceptional chemical, electrical and optical properties. Graphene is very sensitive to any physical changes in its surrounding environment and, inherently, has very low electronic noise. This property of graphene makes it a suitable candidate for sensor applications. The purpose of the work presented in this article is to demonstrate the ability of graphene derivatives to detect toxic organic compounds like phenol and methanol. A novel method for the detection of organic compounds (phenol and methanol) has been introduced in this article. In this method, a change in the photocurrent, as well as electric current, have been used as detection signals to improve the sensor accuracy and selectivity for specific target molecules. A nanoscale electronic device simulator, Quantumwise Atomistix Toolkit (ATK), has been used to simulate graphene nanosheet and armchair graphene nanoribbon based sensors. Devices density of states (DOS), current–voltage curves and photocurrent curves have been calculated with the ATK simulator. In the proximity of target molecules, a significant change in DOS, electric current and photocurrent have been observed. The simulated graphene based structures can be converted into physical sensors to obtain a low cost, small sized, integrated sensing device.

## 1. Introduction

In modern society, the development of a cheap, portable and reliable gas sensor for domestic and industrial environments is of utmost importance. Industrial environment monitoring, for the health and safety of the workforce, demands highly reliable sensors for the detection of toxic compounds [1]. The demand for gas sensors has been increased with the development of the internet of things (IoT). The applications of these sensors include the chemical industry [2,3], food quality assessment [4,5] and the agricultural sector [6,7]. Graphene is a promising candidate for modern gas sensor applications due to its good conductivity [8], good light transmittance [9] and good thermal conductivity [10]. At room temperature, the mobility of charge carriers in graphene remains ballistic for the length of 0.3 µm. A large surface area to volume ratio allows graphene to be exposed to the target molecules more effectively [11]. Inherently, graphene has a very low electronic noise [12], which makes it an astonishing material to detect gases and other related organic compounds. Graphene nanosheet has sp^2^ bonded carbon atoms that are tightly packed to give a honeycomb lattice structure. Graphene can be categorized from a single layer to several stacks of single layers called multilayers. It is mostly known as a zero-gap semiconductor, whose band gap can be tailored according to the application requirement [13,14]. Single layer of graphene (SLG) has numerous unique attributes, especially its band structure. SLG exhibits ambipolar properties (charges can be alternated between electrons and holes based on applied voltages) [15]. All the above-mentioned properties make graphene highly suitable for gas sensor applications. 

A wide range of graphene-based devices are being investigated for the detection of organic compounds and gases. Several advancements in the technology have revolutionized the field of graphene-based sensors and devices. Recently, laser induced graphene paper (LIGP) has emerged as a promising candidate to detect liquids and antibacterial media [16]. Graphite nanoplatelet thin film fiber sensors, with very high sensitivity, have also been fabricated to detect stress/strain and failure of the host composite material [17]. Graphene like materials have been developed to detect volatile organic compounds [18]. Graphene-based flexible, and wearable, gas sensors have been fabricated to detect a wide range of gases and organic chemicals [19].

Moreover, graphene-based gas and organic compound detectors have different working principles. They may include resistive method, micro-electro-mechanical systems (mems) method and field effect transistors (FET) methods. Most of the commercial gas sensors use the resistive method to detect foreign gas particles. The conductivity of the graphene changes with the change in the gas concentration in the environment [20]. The presence of the gas can be measured as a function of change in conductivity of graphene [21]. In FET based devices, drain to source current changes in the presence of the adsorbed target molecules [22,23]. In the case of quality sensitive gas sensors, the operating frequency of the device changes in the presence of a target gas or organic compound [24]. The working principle of the first reported graphene-based gas sensors was also based on the resistive method [12]. That sensor was capable of detecting individual gas and volatile organic molecules. The resistive method based graphene sensors can be easily developed. The absorbed gas, and volatile organic molecules, interact with the graphene surface and change its conductivity by changing the charge carrier concentration. This method provides a high sensitivity to the sensor and even very low concentrations of gases/organic vapors can be detected [25]. The resistive method is being widely used to fabricate sensors to detect gases and organic/inorganic compounds [12].

Furthermore, graphene has a unique electronic band structure that is different from semiconductors, insulators and conductors. It has a conical band structure above and below the Fermi level. These conical band structures are called Dirac cones [26]. Graphene sheets and ribbons have different electronic attributes from each other. Even the electronic properties of armchair and zigzag nanoribbons are dissimilar. Depending on the termination pattern of the edge of the ribbons, their electronic properties change. The bandgap of armchair graphene nanoribbons (AGNRs) changes with the increase in the number of carbon atoms in the ribbon. This bandgap decreases gradually from 3 to 0.75 eV as the number of carbon atoms increases from 20 to 65 atoms, approximately. Whereas, this change for zigzag nanoribbons, is different from AGNRs. For an even number of electrons in the carbon atoms, the change in the bandgap is different compared to that of an odd number of electrons in the carbon atoms. For both cases, the bandgap gradually decreases with an increase in the number of carbon atoms. Doping of boron and nitrogen may be the reason for this change in the bandgap, which changes the ionization energy of the ribbons [27]. Graphene monolayer 2D crystals are fabricated on a large scale by chemical vapor deposition (CVD) [28], and epitaxial growth, on SiC substrates. However, pristine graphene, obtained from these methods, is a gapless semiconductor that limits its applications in nanoelectronics. Theoretical and experimental studies have demonstrated the change in the properties of these nanoribbons compared to those of 2D crystals [29]. Graphene nanoribbons (GNRs) and nanosheets are being used for gas sensor applications [30,31].

Phenol and methanol are toxic and flammable organic compounds that are harmful to human health so it is essential to detect their presence in domestic and industrial environments to avoid serious accidents [32,33]. In this article, phenol and methanol molecules have been detected by using pristine graphene nanosheet and AGNRs. The reason for using pristine graphene for the detection of phenol and methanol is its better response and low detection limit. Even a change by a single atom can be detected. Although graphene-based metal oxide semiconductor sensors are being widely used, but they have a short lifetime, poor selectivity and a high operating temperature [34]. Most of the graphene-based sensors detect the target molecules by a change in its conductivity [20]. The same principle has been used in our work to detect phenol and methanol molecules. This article is an extension to our previously published work [35].

In that paper [35], phenol and methanol molecules have been detected by a change in electric current through a graphene nanosheet. In the current article, a novel mechanism for the detection of phenol and methanol molecules, to improve the sensor selectivity for these target molecules, is proposed. This novel method introduces the change in photocurrent through AGNRs in the proximity of these target molecules. The simulation results demonstrate a significant change in the density of states (DOS), electric current (through graphene nanosheet and AGNRs) and photocurrent (through AGNRs) in the presence of target molecules. These changes in the above-mentioned parameters can be used as the detection signals to develop a physical sensor, based on the graphene nanosheet or AGNR, over a suitable substrate like SiC. The purpose of these simulations is to access the feasibility of graphene nanosheet, or AGNR, based sensors for the detection of phenol and methanol molecules before physical fabrication.

## 2. Materials and Methods

**Figure 1:**
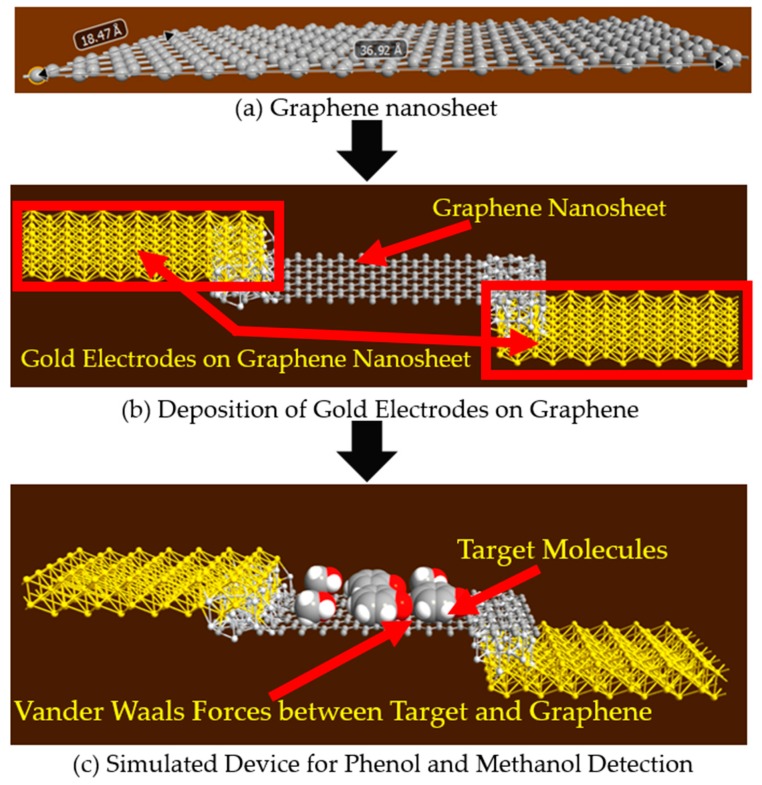
Schematic of the simulated graphene nanosheet based phenol and methanol detector.

Phenol and methanol molecules have been detected with two types of devices. First, graphene nanosheet based structures, with gold electrodes, have been used to detect target molecules, as shown in Figure 1. Secondly, graphene armchair nanoribbon-based structures have been used to detect phenol and methanol molecules. These simulations have been conducted in a nanoscale semiconductor device simulator using, Quantumwise Atomistix Toolkit (ATK), which has a graphical user interface, called Virtual nano lab (VNL), that allows the simulation of a variety of nanoscale devices. This complete software package, with the graphical user interface, is called ATK-VNL. More details about the used simulator will be given in the next section.

### 2.1. Graphene Nanosheet for the Detection of Phenol and Methanol Molecules

In our previously published work [35], graphene nanosheet based structures were used to detect the presence of phenol and methanol molecules. In this study the schematic of the simulated structure is shown in Figure 1. This graphene nanosheet has an approximate length of 37 Å and width of 18 Å, and is simulated with the ATK-VNL, as shown in Figure 1a. In the next step, gold electrodes have been deposited at both edges of the graphene nanosheet, as shown in Figure 1b. In step three, phenol and methanol molecules have been exposed to the surface of the simulated device to detect their presence. Van der Waals forces are acting between the target molecules and the graphene nanosheet, as the target molecules are only a few Angstroms above the surface of the graphene sheet, as shown in Figure 1c.

During the characterization of the simulated device, DC bias voltage is applied to the gold electrodes of the simulated device. Current–voltage (IV) curves have been calculated for four different scenarios: In the absence of target molecules, in the presence of only four phenol molecules, in the presence of only four methanol molecules and in the presence of both molecules (two-phenol and two-methanol molecules). For the same scenarios, four different DOS have also been calculated under zero-bias conditions.

### 2.2. Armchair Graphene Nanoribbon (AGNR) for the Detection of Phenol and Methanol Molecules

**Figure 2:**
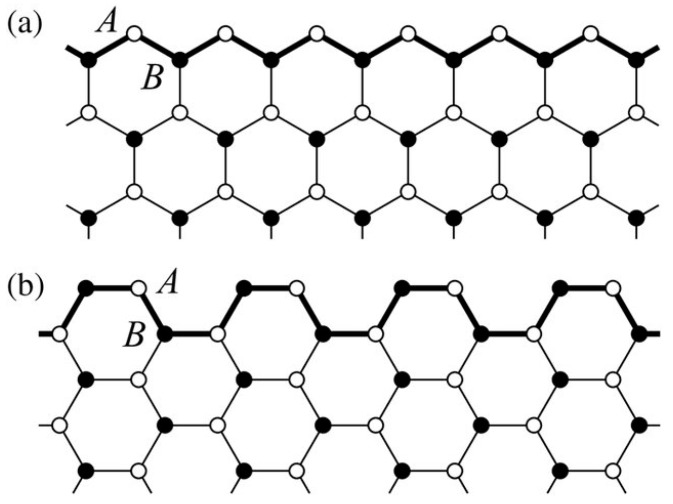
(**a**) Zigzag graphene nanoribbon; (**b**) armchair graphene nanoribbon [36].

This simulated structure is an extension to our previously published work [35]. Termination of infinite graphene layers into finite structures results into two types of nanoribbons [36]. Depending on the termination sequence of the edge, they are categorized into armchair and zigzag nanoribbons, as shown in Figure 2.

**Figure 3:**
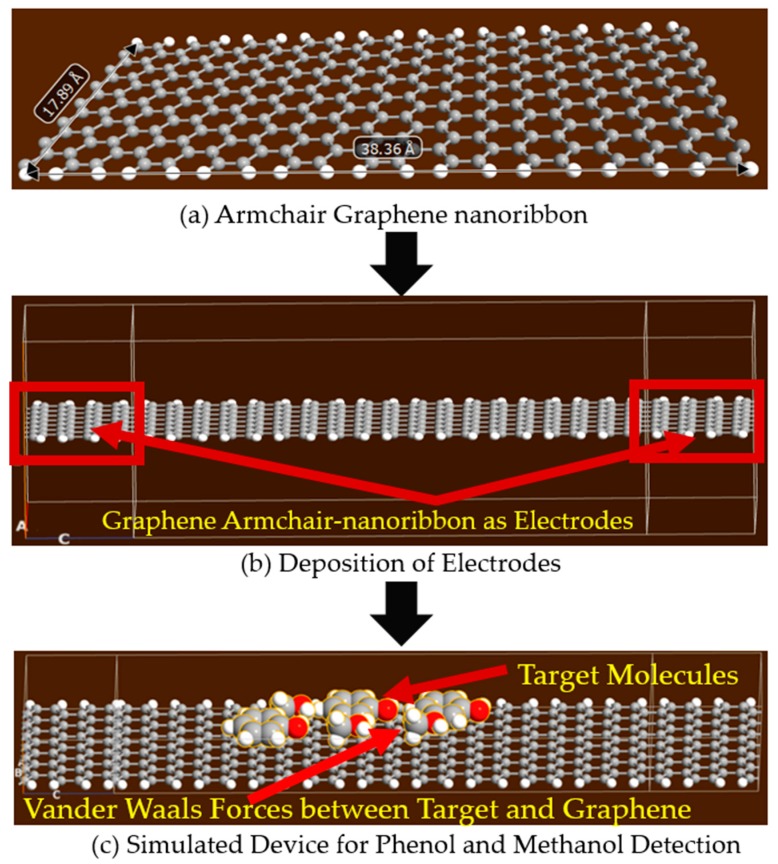
Schematic of the simulated armchair graphene nanoribbon-based phenol and methanol detector.

In Figure 2b, the structure of an armchair graphene nanoribbon is shown. In the simulations, AGNR based structures have been used to detect the presence of phenol and methanol molecules, as shown in Figure 3. Armchair graphene nanoribbons, with width (armchair edge side) of approximately 37 Å and with length (repetition pattern side) of 18 Å, have been simulated with ATK-VNL, as shown in Figure 3a. In the next step, AGNR itself has been chosen as the electrodes (shown within red rectangles) to avoid any Schottky barrier formation at the electrodes, as shown in Figure 3b. This approach reduces the computational time as well. Finally, phenol and methanol molecules have been exposed to the simulated structure, as shown in Figure 3c. The same process described in Section 2.1 has been repeated for the characterization of this device. Device DOS, IV-curves and photocurrent curves have been calculated for four different scenarios.

### 2.3. Methodology for Simulated Graphene Nanosheet and Armchair Graphene Nanoribbon Based Devices

For the simulations of the structures described in Section 2.1 and Section 2.2, an atomic scale semiconductor device simulator, Quantumwise Atomistix Toolkit (ATK) has been used. ATK has a graphical user interface called Virtual Nanolab (VNL). The ATK-VNL software package models the electronic properties of the quantum systems [37]. This software package has different types of in-built calculators that use different methodologies to compute the electronic parameters of the simulated devices. For these simulations, the inbuilt ATK-DFT calculator [38] together with a high-power computing (HPC) environment [39] has been used.

This HPC has 232 computing machines with 1024 GB of total memory. With eight computing nodes, each IV-curve took about one week to calculate in HPC for these experiments. ATK-VNL follows a workflow to simulate electronic devices. In this workflow, first of all the structure of the device is formed using the ATK builder tool, then this simulated structure is sent to the script generator tool that generates the python file. During the script generation, an inbuilt calculator is added into the simulated structure with the desired parameters to be analyzed. The script contains a simulated device, new calculator and density of states (to be analyzed). Finally, the script has been generated as a script.py file. The python code, generated by the scripter can be customized by using the custom scripter tool of ATK-VNL. In the last step, this generated script is run in the job manager. The script can be run on the local machine or on a remote high-power computing machine. After the execution of the job, the output file is obtained (for example, script.nc or script.hdf5). Finally, the results are viewed in the viewer tab. The ATK-DFT in-built calculator was used for the simulations with K-point sampling of K_A_ = 5, K_B_ = 5 and K_C_ = 50.

## 3. Results and Discussions

This section has been divided into two subsections, as two types of devices have been simulated. The density of states (DOS) and IV-curves have been calculated for graphene nanosheet and AGNR based devices.

### 3.1. Density of States and Current–voltage Characteristics Analysis of the Graphene Nanosheet Based Device

**Figure 4:**
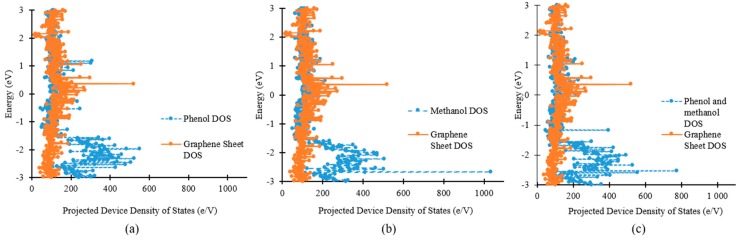
Change in density of states of graphene nanosheet based device in the presence of (**a**) only phenol molecules; (**b**) only methanol molecules; (**c**) both phenol and methanol molecules.

In Figure 4, the projected device density of states (PDDOS) in the presence of phenol, methanol and both phenol and methanol molecules have been shown. In Figure 4a, a noticeable change in the PDDOS of the device could be seen in the presence of phenol molecules (blue lines) compared to that of in the absence of target molecules (orange lines). Exposure of the device to the phenol molecules had introduced many new energy states below the Fermi energy level. At energies ‒1 to ‒3 eV, new energy states with higher values could be observed, compared to the device in the absence of target molecules. This change would definitely affect the current through the graphene nanosheet based device. The effect of methanol molecules on the PDDOS was totally different, as compared to the previous case, as shown in Figure 4b. Many sharp energy states could be observed, below the Fermi level, in the presence of methanol molecules only. These energy states were different from those introduced by phenol molecules.

In the third scenario (Figure 4c), both phenol and methanol molecules were exposed to the graphene nanosheet based device. The change in the PDDOS in this case was totally different from the last two scenarios i.e., Figure 4a,b. Many new energy states could be observed between ‒2 to ‒3 eV in the presence of both phenol and methanol molecules, in Figure 4c. These new states will consequently change the electric current through the device.

**Figure 5:**
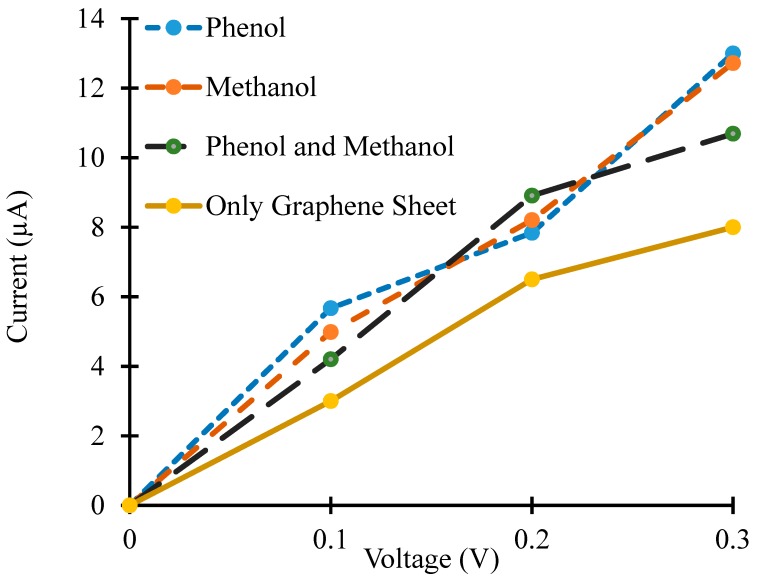
Current–voltage (IV) curves of graphene nanosheet based simulated device in the proximity of phenol and methanol as target molecules.

Figure 5 shows four different IV-curves for four different cases for the simulated device i.e., in the absence of target molecules, in the presence of phenol molecules, in the presence of methanol molecules and in the presence of both phenol and methanol molecules. An increase in the current for the device was observed in the presence of methanol, phenol and both phenol and methanol molecules. The adsorbed target molecules work as acceptors, or donors, for the graphene and change its conductivity [11,40,41]. Adsorbed target molecules were responsible for the change in the charge carrier’s concentration in the graphene nanosheet. If these molecules act as donors for graphene, then they increase its conductivity. Alternatively, the adsorbed molecules reduce the electrical conductivity of graphene if they are acting as acceptors [42]. The simulation results shown in Figure 5 reveal that the target molecules were acting as donors for the graphene nanosheet and changing the concentration of charge carriers in it, but this change in the concentration of charge carriers and conductivity was dissimilar for the different adsorbed molecules (i.e., phenol, methanol and both phenol and methanol molecules).

Moreover, the difference in the electric current values is not very significant for different target molecules in the simulations. This could be due to a lesser concentration of target molecules that are exposed to the graphene nanosheet. The concentration of target molecules has been kept low (four molecules in each case) to minimize the computational time of the simulations. Even with this lesser concentration of target molecules, each IV-curve took more than one week to calculate in HPC. This difference in current values would be more obvious with an increased concentration of target molecules.

### 3.2. Density of States and Current–Voltage Characteristics Analysis of Armchair Graphene Nanoribbon Based Device

**Figure 6:**
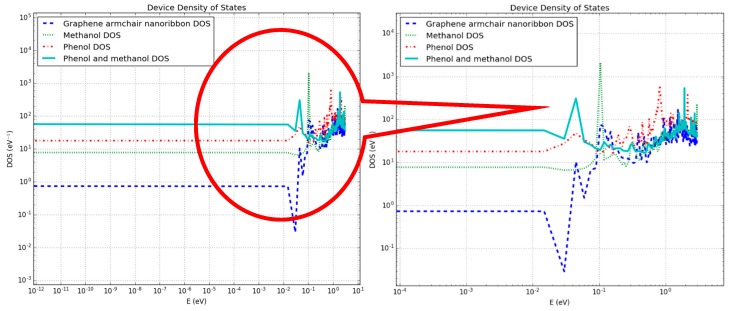
Change in density of states of an armchair graphene nanoribbon-based device in the proximity of target molecules where the x- and y-axis are in logarithmic scales (Right figure is a magnified view of left figure).

In another set of experiments, armchair graphene nanoribbon (AGNR) of approximately the same dimensions as the graphene nanosheet (dimensions are given in Figure 1 and Figure 3) were used for the detection of phenol and methanol molecules. The device density of states (DOS), IV-curves and photocurrent curves were calculated for the same target molecules. The purpose of detecting the same target molecules with AGNR was to analyze which graphene-based material was more sensitive and suitable for the development of a physical sensor. In Figure 6, energy vs. density of states was shown on logarithmic scales. In the magnified view (right image) of Figure 6, it can be clearly seen that the DOS of AGNR (dark blue dotted line), in the absence of target molecules, was significantly different from all other three DOS curves. In Figure 6, the green dotted line, red dotted line and solid greenish blue line are representing the DOS of the device in the proximity of methanol, phenol and both methanol and phenol molecules, respectively (also see the legend). It can be observed in Figure 6 that in the presence of methanol molecules, the abrupt spikes in energy states were not present between energy levels of 10^‒2^ to 10^‒1^ eV, as compared to that of the device in the absence of target molecules.

Moreover, the comparison of energy states for the rest of the energy window between the device in the absence of target molecules and the device in the presence of methanol shows that they were different from each other. The presence of methanol molecules had changed the DOS of the pristine AGNR. This figure shows that DOS curves of the AGNR based device in the presence of only phenol, and both phenol and methanol, molecules had changed the energy states differently. This change in DOS of the device would definitely influence the electric current through the device in the proximity of these target molecules.

**Figure 7:**
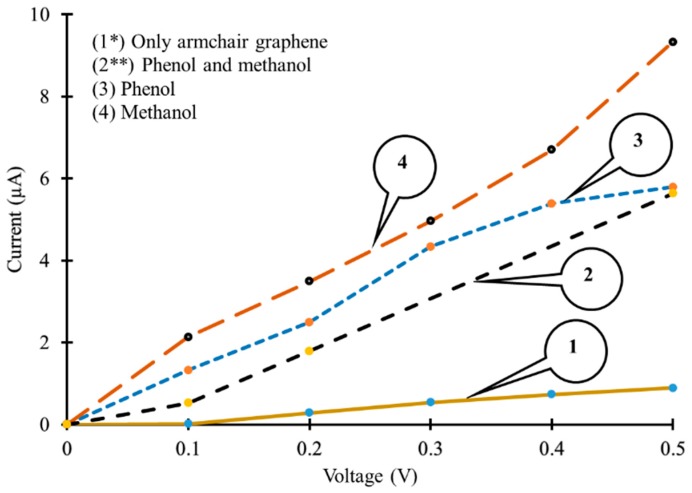
Current–voltage (IV) curves of armchair graphene nanoribbon based simulated device (1*) without target molecules (current axis ×10^‒18^ to get actual values); (2**) with phenol and methanol molecules (current axis ×10^‒13^ to get actual values); (3) with phenol molecules; (4) with methanol molecules.

Furthermore, Figure 7 shows the change in IV-curves of AGNR based device in the presence of phenol, methanol and both phenol and methanol molecules as target molecules. Simulation results reveal that in the absence of target molecules, a very low current in the range of 10^‒20^ to 10^‒19^ µA flowed through the device, as shown in Figure 7 (curve 1). Exposure of the AGNR based device to both phenol and methanol molecules resulted in an increase in the electric current through the device. The range of the electric current through the device in this case was of 10^‒14^ to 10^‒13^ µA, as shown in Figure 7 (curve 2). The influence of the phenol and methanol molecules seemed to be like donors of charge carriers for AGNR, due to which an increase in the electric current, with reference to curve 1, was observed in the presence of both phenol and methanol molecules.

Furthermore, it was observed that the exposure of an AGNR based device to phenol molecules only, significantly increased the flow of the electric current through the device as shown in Figure 7 (curve 3). Phenol molecules may have acted as donors of charge carriers for AGNR, due to which a significant increase in the electric current through the device, with reference to curves 1 and 2, was observed, as shown in Figure 7. The range of current in this case was between 1.325 to 5.788 µA approximately. A similar increase in current through the device was observed in the presence of only methanol molecules. In this case, methanol molecules might have acted like donors for AGNR but the range of current was between 2.131 to 9.323 µA approximately, as shown in Figure 7 (curve 4).

#### PhotoCurrent Analysis of an Armchair Graphene Nanoribbon Based Device in the Proximity of Different Target Molecules

In order to improve the sensor reliability and selectivity for specific target molecules, more than one detection mechanism can be used. In Section 3.2, the change in electric current in the presence of target molecules was used as a signature to detect them. In this sub-section, change in the photocurrent in the presence of target molecules was used as a detection signal, along with a change in electric current, to enhance the device selectivity for specific molecules. The AGNR based device has been illuminated by AM1.5 solar spectrum obtained from [43], where AM1.5 stands for the air mass coefficient that is used to characterize the performance of solar cells, for terrestrial power-generating panels, under standardized conditions. In these simulations, photon energies have been kept between 0 to 3 eV and the resultant photocurrent was calculated. Generation of a photocurrent in AGNR was observed under the above-mentioned illumination conditions in the simulator. This kind of first principles simulation has been reported in the literature for solar cell devices [44]. The same principle was implied for the AGNR sensor.

**Figure 8:**
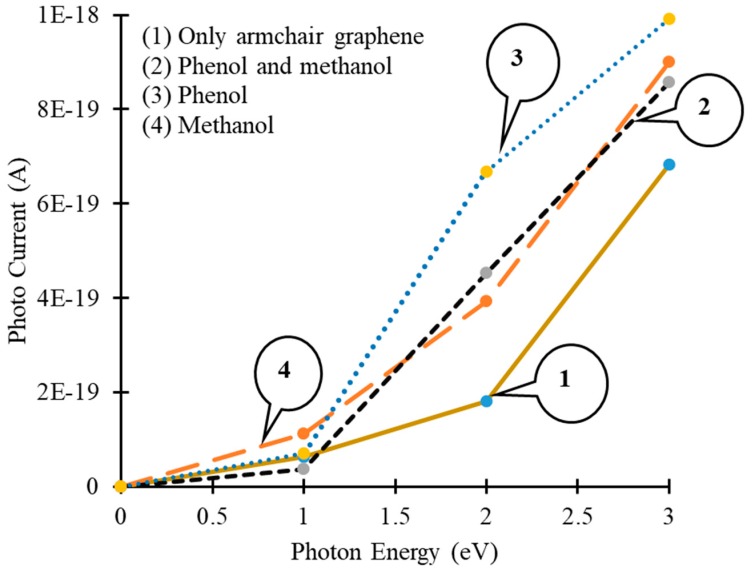
Photon energy vs. photocurrent curves of an armchair graphene nanoribbon device (1) without target molecules; (2) with phenol and methanol molecules; (3) with phenol molecules and (4) with methanol molecules.

During the physical characterization process, the AGNR based device could be exposed to an artificial light source (a light bulb or LED) to generate a photocurrent. Then the change in photocurrent, in the presence of different target molecules, could be used as a signature signal to detect specific molecules. The AGNR based device was illuminated with photons, having energies between 0 to 3 eV, and the change in photocurrent was calculated for different target molecules, as shown in Figure 8.

Moreover, the results show that with an increase in photon energies, the generation of photocurrent also increases, but the change in photocurrent, for different target molecules, was dissimilar, as shown in Figure 8. During the physical characterization of the graphene-based sensor, the change in the electric current, as well as the photocurrent, can be used as detection signals. Afterwards, these signals can be fed to, and stored in, a micro-controller as a signature signal for the detection of a specific target. Finally, an inbuilt light source can be installed for the illumination of the AGNR to get a photocurrent in a physically fabricated device.

However, a very low photocurrent has been observed during the simulations in AGNR as shown in Figure 8. The simulation of light absorption in a single layer of graphene, is still a key challenge, although graphene based optoelectronic devices, with high internal quantum efficiencies, have been reported for physically fabricated devices. Due to the dissimilarities of the behavior of traditional semiconductors and graphene, in the generation and transportation of charge carriers, high-bandwidth and high internal quantum efficiency values have been reported for graphene based experimental devices [45], but it is not an easy task to reproduce the same results, by simulations, due to limitations of the available simulators in relation to the material’s parameters and other complex phenomena. Hot carriers play a crucial role in the generation of photocurrents in optoelectronic devices, but the mechanism involved in this process has not been fully determined yet [46]. The key challenge is to resolve the differences, between the theoretical material properties and the experimental results, to achieve more accurate simulation-based results.

However, with heavy chemical doping, the absorption in a single layer of graphene can be increased physically up to 40%. The generation of a photocurrent also depends on the interaction of graphene plasmons with surface polar phonons of insulating substrates, like SiO2. This hybrid plasmon–phonon mode, between graphene plasmons and its substrate’s polar phonons, can be excited in the mid-infrared region under s-polarization. In this interaction, the phonon’s and electron’s temperature are raised and this increased temperature changes the electrical conductivity and increases the photocurrent in graphene. Due to the limitations of the simulator in controlling the polarization of photons and the activation of the plasmon-phonon coupling mode (in the case of using an insulating substrate for AGNR), high values of photocurrent could not be achieved in the simulations. However, in physically fabricated graphene-based devices, high photocurrents can be achieved by tuning intrinsic plasmons and activating the hybrid plasmon–phonon mode [47].

Moreover, we are planning to conduct experiments based on differential measurements to study the effect of light on the generation of photocurrents in graphene-based devices. In these experiments two graphene strips will be placed in parallel within a controlled environment. In the experimental setup, one strip will be illuminated with a suitable light source and the other one will be kept in darkness. After that, the differential signal will be acquired from these two graphene strips with the help of a locally developed amplifier [48] to get rid of noise and acquire the information signal (photocurrent).

Pristine graphene nanosheet and AGNRs were used to detect phenol and methanol molecules in our simulations. However, in practice, it is not possible to avoid defects in the large-scale fabrication process of graphene-based devices. Defects in graphene change its electronic properties. Sometimes, defects can contribute to the introduction of beneficial effects in graphene-based devices that can be utilized for different device applications. More significant effects of defects are self-heating and a degraded performance of the device [49].

## 4. Conclusions

The purpose of the work that has been presented in this article is to access the feasibility of the graphene nanosheet and armchair graphene nanoribbon to detect phenol and methanol molecules prior to the physical fabrication of a sensor. The simulation results showed that both phenol and methanol could be detected with a graphene nanosheet and armchair nanoribbons, but the sensitivity of a graphene nanosheet is very low for the detection of the desired target molecules. The change in the electric current of the graphene nanosheet is very small in the presence of phenol and methanol as target molecules. In a low concentration of these target molecules, it is very difficult to differentiate between the presence of phenol, methanol and both phenol and methanol molecules. Whereas, the selectivity and sensitivity of armchair graphene nanoribbon, to detect phenol and methanol molecules, is better than the graphene nanosheet. A significant difference between the IV-curves of AGNR based device has been observed in the presence of phenol and methanol as target molecules. Furthermore, a change in the photocurrent through AGNR, in the presence of the above-mentioned target molecules, was also calculated, although, the values of the photo generated current were very small in the simulated devices. By controlling the polarization of photons and activating the plasmon-photon coupling mode (after selecting a suitable insulating substrate for AGNR), higher values of photocurrent could be physically achieved. In order to improve the sensor selectivity and accuracy, changes in the electric current, as well as the photocurrent, are suggested as signature signals for the detection of phenol and methanol molecules. These simulations revealed that AGNR is a better choice for the detection of phenol and methanol molecules as the change in IV-curves, in the presence of target molecules, is more obvious as compared to that of graphene nanosheet. The future intention is to develop a physical sensor based on AGNR to detect phenol and methanol molecules. Changes in the electric current and photocurrent, in the presence of specific target molecules, will be used as a detection signal for the AGNR based physical sensor.
